# Use of anesthesia dramatically alters the oral glucose tolerance and insulin secretion in C57Bl/6 mice

**DOI:** 10.14814/phy2.12824

**Published:** 2016-06-02

**Authors:** Johanne A. Windeløv, Jens Pedersen, Jens J. Holst

**Affiliations:** ^1^ Novo Nordisk Foundation Center for Basic Metabolic ResearchTranslational Metabolic Physiology and Department of Biomedical SciencesUniversity of CopenhagenCopenhagenDenmark

**Keywords:** Anesthesia, metabolic test, oral glucose tolerance test

## Abstract

Evaluation of the impact of anesthesia on oral glucose tolerance in mice. Anesthesia is often used when performing OGTT in mice to avoid the stress of gavage and blood sampling, although anesthesia may influence gastrointestinal motility, blood glucose, and plasma insulin dynamics. C57Bl/6 mice were anesthetized using the following commonly used regimens: (1) hypnorm/midazolam repetitive or single injection; (2) ketamine/xylazine; (3) isoflurane; (4) pentobarbital; and (5) A saline injected, nonanesthetized group. Oral glucose was administered at time 0 min and blood glucose measured in the time frame −15 to +150 min. Plasma insulin concentration was measured at time 0 and 20 min. All four anesthetic regimens resulted in impaired glucose tolerance compared to saline/no anesthesia. (1) hypnorm/midazolam increased insulin concentrations and caused an altered glucose tolerance; (2) ketamine/xylazine lowered insulin responses and resulted in severe hyperglycemia throughout the experiment; (3) isoflurane did not only alter the insulin secretion but also resulted in severe hyperglycemia; (4) pentobarbital resulted in both increased insulin secretion and impaired glucose tolerance. All four anesthetic regimens altered the oral glucose tolerance, and we conclude that anesthesia should not be used when performing metabolic studies in mice.

## Introduction

The oral glucose tolerance test (OGTT) is a widely used experimental procedure for metabolic studies in mice. Although the NIH mouse Metabolic Phenotyping Center Consortium has provided a standard operating procedure for performing glucose tests in mice (Ayala et al. 2010), most studies differ significantly with respect to parameters such as anesthesia, fasting time, glucose dose, gavage material, time points for blood glucose (BG) measurements, and time points for blood sampling for hormonal analysis. This clearly jeopardizes comparisons across studies. In this study, we investigated the impact of four anesthetics for rodents on oral glucose tolerance and insulin responses in mice. It is known that anesthetics may influence gastric emptying, intestinal motility, BG and insulin (Goldfine and Arieff 1979; Anderson et al. 2002; Inada et al. 2004; Pomplun et al. 2004; Brown et al. 2005; Saha et al. 2005; Rodrigues et al. 2006; Zuurbier et al. 2014), but anesthesia is still used for OGTTs (Pacini et al. 2013) to avoid the stress of the gavage and blood sampling. Previous studies in rodents, not including oral glucose tolerance tests, have shown that: (1) the hypnorm/midazolam combination (Hyp/Mid) causes little alteration in BG or insulin (Zuurbier et al. 2014), whereas great increases in both BG and insulin may be seen after administration of only hypnorm (Johansen et al. 1994); (2) the ketamine/xylazine combination (Ket/Xyl) increases BG and inhibits insulin secretion (Goldfine and Arieff 1979; Pomplun et al. 2004; Brown et al. 2005; Saha et al. 2005; Rodrigues et al. 2006); (3) the volatile agent isoflurane increases BG and some have noted a decrease in insulin concentration (Pomplun et al. 2004; Saha et al. 2005; Zuurbier et al. 2008, 2014), and in humans, isoflurane impairs glucose clearance and decreases insulin secretion during an IVGTT (Intravenous glucose tolerance test) (Tanaka et al. 2005); (4) pentobarbital does not result in increased BG, but increases insulin concentration (Johansen et al. 1994; Saha et al. 2005; Zuurbier et al. 2008, 2014). The inbred C57Bl/6 mouse strain is commonly used for metabolic research as many genetically modified mice strains are bred on this background. Using this strain, we investigated the impact of four anesthetics during an OGTT measuring BG and plasma insulin concentration at times 0 and 20 min.

## Methods and Materials

### Mice

Female mice age 10–11 weeks, C57BL/6, were purchased at Charles River Laboratories International Inc. Mice were housed in groups of 6–8 mice in individually ventilated cages under a light cycle of 12 h (lights on 6 am to 6 pm) with ad libitum access to normal chow and water. The experiment was conducted in accordance with institutional guidelines (EMED, University of Copenhagen) and approved by the Animal Experiments Inspectorate, Danish Ministry of Environment and Food (license no. 2013‐15‐2934‐00833).

### Anesthetics

We used (1) hypnorm (fluanisone/fentanyl) in combination with midazolam (Premixed Hypnorm/Midazolam, Department of Experimental Medicine, KU, 0.79 mg/kg fentanyl citrate + 25 mg/kg fluanisone + 12.5 mg/kg midazolam) given IP, either with a single bolus, followed by ½ dose every 20 min Repetitive injections of hypnorm/midazolam (Hyp/Mid Rep) for full surgical anesthesia, or as a single bolus injection Single injection of hypnorm/midazolam (Hyp/Mid Single); (2) ketamine (Ketaminol^®^, Intervet, Merck & CO., New Jersey, catalog no. 511485, dose 100 mg/kg) in combination with isotonic saline (0.9% NaCl) and xylazine (Rompun^®^vet, Bayer AG, Leverkusen, Germany, catalog no. 148999, dose 10 mg/kg) given IP as a start dose followed by a ½ dose supplement after 40 min; (3) pentobarbital (Pentobarbital, ref: 1347007, Department of Experimental Medicine, KU, dose 40 mg/kg) given IP after premedication with analgesics (lidocain; Lidokain, Sygehus Apotekerne I Danmark, ref: 742445 dose 4 mg/kg); (4) isoflurane (Forane, Baxter Healthcare Corporation, Deerfield, Illinois, catalog no. 1001936060) given in a low concentration (1%) after induction with a higher concentration (3%). A control group was injected IP with isotonic saline (0.9% NaCl) with a volume of 0.2 mL. The anesthesia confirmed by loss of the pedal withdrawal reflex following pinching with a forceps between the toes of the right hind foot, just before oral gavaging.

### Oral glucose tolerance test

Mice were fasted overnight (16–18 h) with free access to water. Mice were anesthetized (IP injection or gas form) or given a saline injection (IP injection) at time −15 min before oral gavage of glucose, 50% w/v solution, in a dose corresponding to 2 g glucose per kg bodyweight. Control mice were returned to their cages following oral gavage and were allowed to move around between the blood sampling. For pentobarbital injected animals, a lidocaine IP injection was given at time −20 min in the area where pentobarbital was to be injected at time −15 min to minimize the pain induced by pentobarbital. BG was measured after tail tip punctures, at times −15, 0, 20, 40, 60, 90, 120, and 150 min after oral glucose load using a handheld plasma calibrated glucometer (Accu‐check compact plus, F.Hoffmann‐La Roche AG, Bazel, Switzerland). Blood samples (75 *μ*L) were drawn from the retrobulbar plexus at time 0 and 20 min using EDTA coated capillary tubes (Vitrex Medical, Herlev, Denmark, ref 164213), and transferred to chilled Eppendorf tubes. Blood samples were centrifuged at 2800 *g* at 4°C for 20 min and plasma was transferred to new tubes and immediately frozen on dry ice. Insulin was measured using an insulin ELISA (Mercodia AB, Uppsala, Sweden, catalog no. 10‐1247‐10) according to manufacturers’ protocol; lower detection limit was 200 ng/L.

### Statistics

Data are presented as means ± SEM. Graphs and statistical analysis were calculated using GraphPad Prism 5 software using two‐way analysis of variance (ANOVA) repeated measurements (RM) comparing all five groups followed by Bonferroni post hoc test compared to saline group for repeated measurements, one‐way ANOVA comparing all five groups followed by Bonferroni post hoc test compared to saline group, for single measurements, and incremental Area under the curve (iAUC) was calculated using the trapezoidal rule with baseline as the time 0 min value.

## Results

### Hypnorm/midazolam

**Figure 1:**
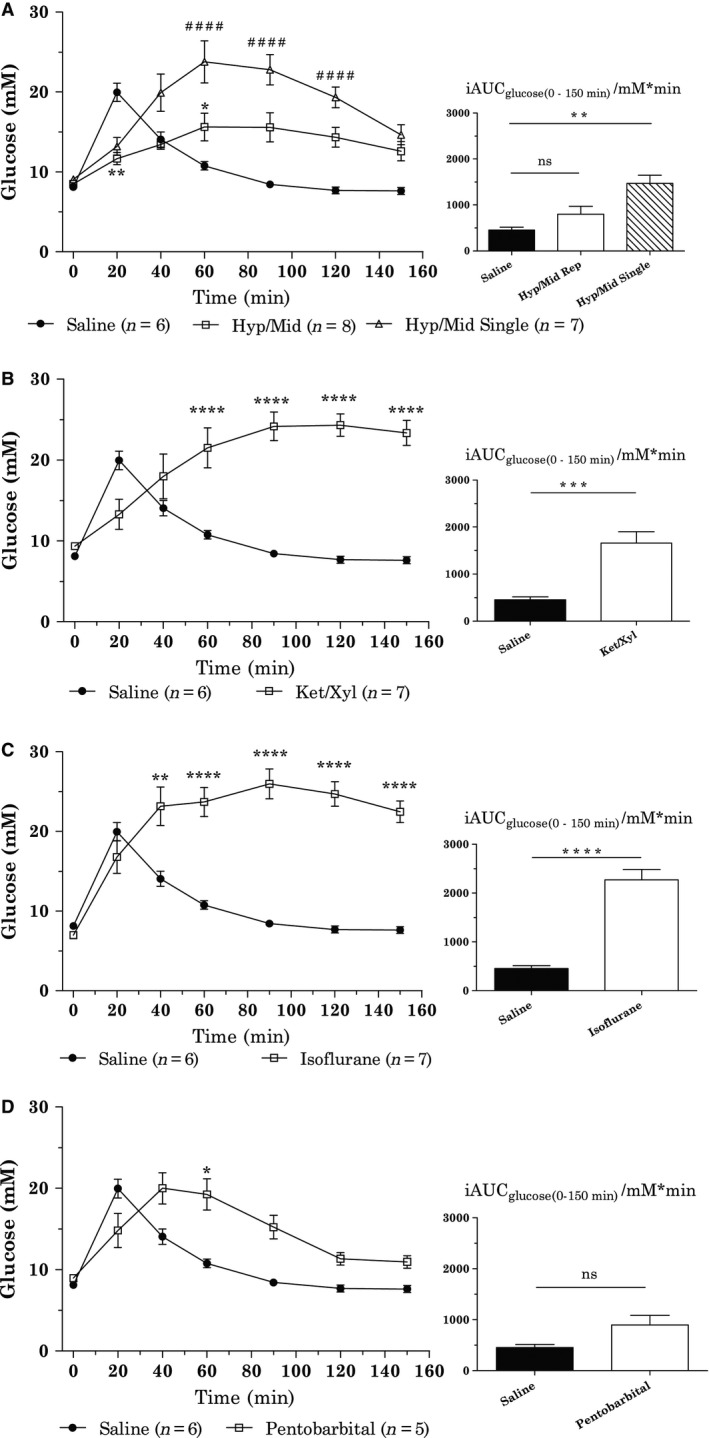
Impact of anesthetics on blood glucose concentration after oral glucose load presented as individually graphs comparing one anesthetic group with the control group (saline), statistics are calculated as two‐way ANOVA RM with all five groups. Saline *n* = 6, Hyp/mid Rep *n* = 8, Hyp/mid single *n* = 7, ket/xyl *n* = 7, isoflurane *n* = 7, and pentobarbital *n* = 5. (A) Saline versus Hyp/Mid Rep and Hyp/Mid Single, (B) Saline versus Ket/Xyl, (C) Saline versus isoflurane, (D) Saline versus pentobarbital (and lidocaine). Inserts: iAUC time 0–150 min. *P < 0.05; **P < 0.01; ***P < 0.001; ****/^# # # #^ *P* < 0.0001 all compared to saline for a given anesthetic at given time (calculated by RM two‐way ANOVA with Bonferroni post hoc test) or for iAUC (one‐way ANOVA with Bonferroni post hoc test). All data are shown as mean ± SEM.

**Figure 2:**
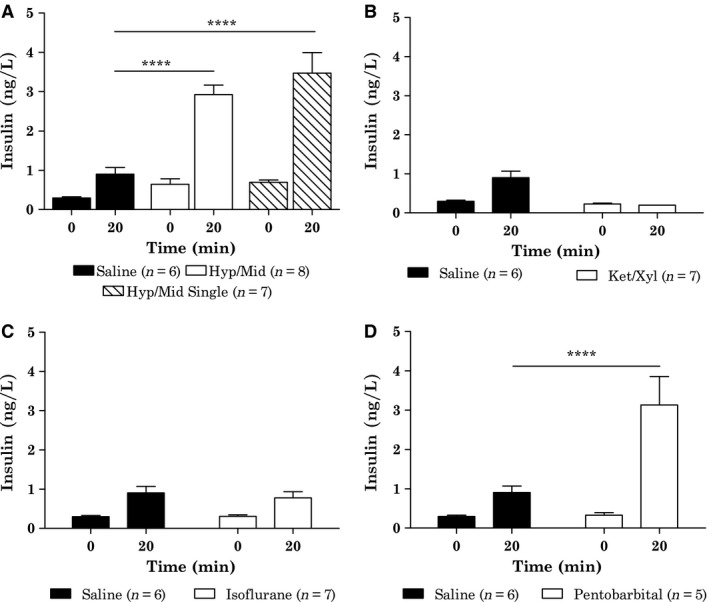
Impact of anesthetics on insulin secretion at time 0 and 20 min after oral glucose load presented as individually graphs comparing one anesthetic group with the control group (saline), statistics are calculated as two‐way ANOVA RM with all five groups. Saline *n* = 6, Hyp/mid Rep *n* = 8, Hyp/mid single *n* = 7, ket/xyl *n* = 7, isoflurane *n* = 7, and pentobarbital *n* = 5. (A) Saline versus Hyp/Mid Rep and Hyp/Mid Single, (B) Saline versus Ket/Xyl, (C) Saline versus isoflurane, (D) Saline versus pentobarbital (and lidocaine). ****P < 0.0001 all compared to saline for a given anesthetic (calculated by RM two‐way ANOVA with Bonferroni post hoc test). All data are shown as mean ± SEM.

Hypnorm/midazolam (Hyp/Mid) combination was applied as repetitive injections (Hyp/Mid Rep) (full surgical anesthesia) or as a single injection at time −15 min (Hyp/Mid Single). Using a single injection of Hyp/Mid, animals had regained reflexes around time 40 min. Both variants of anesthesia resulted in dramatically altered oral glucose tolerance, with the Hyp/Mid Single showing greater impairment, see Figure 1A. The incremental AUC (iAUC) for Hyp/Mid Single was significantly different from Saline (*P*_iAUC_ < 0.01), whereas the iAUC was similar between saline and Hyp/Mid Rep. However, the characteristics of the time‐glucose concentration curve of Hyp/Mid Rep differed significantly compared to the saline group (*P*_20 min_ < 0.01, *P*_60 min_ < 0.05). Insulin concentration at time 20 min after oral glucose load was threefold higher for both Hyp/Mid Rep and Hyp/Mid Single (both *P* < 0.0001) compared to saline, see Figure 2A. The insulin responses obtained during the two variants of Hyp/Mid dosing were similar. There was no difference in blood glucose before anesthesia (time −15 min) between any groups (data not shown).

### Ketamine/xylazine

The ketamine/xylazine (Ket/Xyl) combination also altered the oral glucose tolerance compared to saline (*P*_iAUC_ < 0.001), see Figure 1B. The BG level for Ket/Xyl tended to be lower at time 20 min (*P* > 0.05), but showed a continuous increase and was significantly increased in the time interval 60–150 min (*P* < 0.0001). The insulin concentration of Ket/Xyl injected animals was not increased from time 0–20 min as normally seen after oral glucose load, see Figure 2B. The insulin measurements, for both time 0 and 20 min for Ket/Xyl samples, were around lower detection limit (200 ng/L). There was no difference in blood glucose before anesthesia (time −15 min) between the groups (data not shown).

### Isoflurane

Isoflurane was given continuously at a low concentration (1.0–1.5%) to maintain minimal sedation after induction with a higher concentration (3%). BG concentration was comparable with the saline group in the time interval 0–20 min, but continued to increase with a maximum concentration at time 90 min and was significantly increased in the time interval 40–150 min (*P* < 0.01 to *P* < 0.0001) resulting in dramatically increased incremental AUC (*P*_iAUC_ < 0.0001), see Figure 1C. There was no significant difference in insulin response between saline and isoflurane, see Figure 2C. There was no difference in blood glucose before anesthesia (time −15 min) between the groups (data not shown).

### Pentobarbital

Pentobarbital was given at time −15 min after administration of lidocaine (given at time −20 min) due to the tissue irritation effects of pentobarbital. BG at time −15 min was therefore not measured. BG concentration was comparable with the saline group in the time interval 0–20 min, but continued to increase with a maximum concentration at time 40 min and was significantly increased at time 60 min (*P* < 0.05) although it did not result in an impaired glucose clearance as estimated from iAUCs (*P*_iAUC_ > 0.05), see Figure 1D. Insulin concentration at time 20 min after oral glucose load was threefold higher with pentobarbital (*P* < 0.001) compared to saline.

## Discussion

### Hypnorm/midazolam

Both dosage schemes of Hyp/Mid induced an impaired oral glucose tolerance and a more than threefold increased insulin secretion at time 20 min. A study by Inada et al. showed that midazolam alone decreases gastric emptying and gastrointestinal transit in mice (Inada et al. 2004). Johansen et al. investigated the impact of hypnorm on BG and insulin in rats and showed both increased BG and insulin in fed rats (Johansen et al. 1994). A study by Zuurbier et al.(Zuurbier et al. 2014) showed a significant decrease in blood glucose levels in C57Bl/6 mice both at 30 and 60 min after receiving fentanyl–fluanisone–midazolam, the same compounds as in our Hyp/Mid group. Insulin concentrations were not measured at 30 min, but were similar to control animals at 60 min. We speculate that the significant decrease in blood glucose at 30 min could reflect a relative higher secretion of insulin compared to the blood glucose as we see in our study.

### Ketamine/xylazine

Ket/Xyl induced an impaired glucose tolerance and seems to inhibit glucose‐induced insulin secretion at time 20 min. Previous studies have found similar results showing that Ket/Xyl induces increased BG and decreased insulin secretion in both rats and mice (Pomplun et al. 2004; Brown et al. 2005; Saha et al. 2005; Rodrigues et al. 2006). A study by Goldfine et al. shows an absolute inhibition of glucose‐induced insulin secretion by xylazine, by investigating the insulin secretion after iv glucose administration in dogs (Goldfine and Arieff 1979). In a rat study by Saha et al., ketamine alone did not alter BG levels (Saha et al. 2005), and they concluded that xylazine must be the BG increasing factor. Saha et al. also found an impact on insulin levels in rats after administration of Ket/Xyl, but they did not evaluate the impact of ketamine alone on insulin levels.

### Isoflurane

Low‐flow administration of the volatile anesthesia isoflurane induces an impaired glucose tolerance, although there was no difference in insulin secretion compared to the saline group. The impact of isoflurane has previously been investigated and showed that isoflurane alone induced a significant increase in BG concentration in both fasted and fed rats and mice (Pomplun et al. 2004; Saha et al. 2005; Zuurbier et al. 2008, 2014). Regarding the insulin responses, others have seen either no change or decreases after isoflurane administration (Zuurbier et al. 2008, 2014).

### Pentobarbital

Pentobarbital induces an impaired glucose tolerance and the insulin secretion is altered with a threefold increased insulin concentration at time 20 min. The impact of pentobarbital on BG has previously been investigated in mice and rats, and all studies showed that pentobarbital did not increase blood glucose (Johansen et al. 1994; Saha et al. 2005; Zuurbier et al. 2008, 2014). In two of these studies insulin levels increased in fed rats (Johansen et al. 1994; Zuurbier et al. 2008).

## Conclusion

This study was performed in female mice, but we speculate that the effects observed in this study would be more pronounced in male mice, as it is well established that insulin sensitivity is decreased in male compared to females (Yokomizo et al. 2014). The nonanesthetized mice were allowed to move freely between the blood sampling. However, we observed that the mice generally were inactive between the blood samplings, and therefore consider the possible muscular uptake of glucose, although not measured, to be small. Nevertheless, it was probably bigger than that of the anesthetized animals, and therefore may contribute to some of some of the differences in glucose excursions observed in this study. It is well established that the motility of the gastrointestinal tract is affected by the use of anesthetic agents (Anderson et al. 2002; Inada et al. 2004), and this effect contributes probably also observed effect on the oral glucose tolerance.

In conclusion, this study clearly shows that the use of all four tested forms of anesthesia has an effect on apparent glucose metabolism as seen by altered oral glucose tolerance, and for some, also an altered insulin concentration. The use of anesthetics for metabolic studies in mice is therefore likely to give highly aberrant results and anesthetics should not be used when evaluating oral glucose tolerance in mice.

## Conflict of Interest

None declared.
